# Implications of Schwann Cells Biomechanics and Mechanosensitivity for Peripheral Nervous System Physiology and Pathophysiology

**DOI:** 10.3389/fnmol.2017.00345

**Published:** 2017-10-25

**Authors:** Gonzalo Rosso, Peter Young, Victor Shahin

**Affiliations:** ^1^Institute of Physiology II, University of Münster, Münster, Germany; ^2^Department of Sleep Medicine and Neuromuscular Disorders, University of Münster, Münster, Germany

**Keywords:** peripheral nervous system (PNS) development and regeneration, PMP22-associated PNS neuropathies, Schwann cells, mechanosensitivity, mechanical interactions between cells and the extracellular matrix

## Abstract

The presence of bones around the central nervous system (CNS) provides it with highly effective physiologically crucial mechanical protection. The peripheral nervous system (PNS), in contrast, lacks this barrier. Consequently, the long held belief is that the PNS is mechanically vulnerable. On the other hand, the PNS is exposed to a variety of physiological mechanical stresses during regular daily activities. This fact prompts us to question the dogma of PNS mechanical vulnerability. As a matter of fact, impaired mechanics of PNS nerves is associated with neuropathies with the liability to mechanical stresses paralleled by significant impairment of PNS physiological functions. Our recent biomechanical integrity investigations on nerve fibers from wild-type and neuropathic mice lend strong support in favor of natural mechanical protection of the PNS and demonstrate a key role of Schwann cells (SCs) therein. Moreover, recent works point out that SCs can sense mechanical properties of their microenvironment and the evidence is growing that SCs mechanosensitivity is important for PNS development and myelination. Hence, SCs exhibit mechanical strength necessary for PNS mechanoprotection as well as mechanosensitivity necessary for PNS development and myelination. This mini review reflects on the intriguing dual ability of SCs and implications for PNS physiology and pathophysiology.

## Introduction

The general assumption that the peripheral nervous system (PNS) is prone to mechanical injuries is based on the absence of a formidable physical barrier in the PNS that presents the skull and bony vertebrae in the central nervous system (CNS). Lack of mechanical protection or mechanical strength, however, should impede the physiological activities of the PNS. Take for example the primary nerve function that is the propagation of action potentials. Conduction velocity is used as a criterion to classify nerves. While some nerves exhibit a conduction velocity as low as 0.1 ms^−1^ others exhibit a very high velocity up to 100 ms^−1^ which is of paramount importance for vital physiological responses (Debanne, 2004). The presence of a myelin sheath around nerve fibers is one of the critical factors ensuring a high conduction velocity. Another critical factor is the axon caliber; the larger the faster. Consequently, liability to mechanical compression would substantially slow down the conduction velocity or induce conduction block and the resulting pathophysiological consequences would be severe. Hence, the assumption that the PNS lacks mechanical strength is somewhat shaky and should be reconsidered. The question is pertinent as to whether major constituents of the PNS provide it with natural mechanical strength. Likely candidates are several tissues and extracellular matrices (ECMs) within PNS nerves (Colognato et al., 2005) which may offer some mechanical support. In the PNS, every individual nerve fiber is wrapped in a protective sheath known as the endoneurium. In addition, fibers are bundled in fascicles possessing a protective lining termed perineurium. Fascicles in turn are joined together with a blood supply and fatty tissue within yet another layer known as the epineurium. Unlike oligodendrocytes and their associated axons in the CNS, Schwann cells (SCs) and axons in the PNS are enveloped in a basal lamina, a specialized form of ECM (Colognato et al., 2005). The fairly thin basal lamina is mainly secreted by SCs (Obremski et al., 1993). Different roles have been proposed for the basal lamina including SC proliferation, survival, migration and myelination (Colognato et al., 2005; Court et al., 2006; Chernousov et al., 2008; Colognato and Tzvetanova, 2011; Pereira et al., 2012). The role of the basal lamina in providing mechanical strength to PNS nerves has recently been demonstrated (Rosso et al., 2014) and will be discussed in the present review. Besides, recent observations *in vitro* demonstrate that SCs are capable of sensing the mechanical stiffness of their ECM protein-coated substrate (Rosso et al., 2017). Mechanosensitivity strongly impacts SCs shape, migration, adhesion and mechanics (Rosso et al., 2017) and is thus assumed to play key roles in PNS development, maintenance, myelination and regeneration as discussed here.

## Mechanosensitivity

It should be noted that cells interactions with ECMs is inherently not only biochemical but also strongly mechanical (Discher et al., 2005). There is overwhelming evidence for the importance of mechanical interactions between the ECM and cells as shown for diverse cell types (Even-Ram et al., 2006; Discher et al., 2009; Franze, 2013). Tissue cells are naturally not suspended in fluid but anchored which is essential for their viability. Anchorage on the other hand exposes cells to mechanical forces imposed upon them by the physical properties of their environment, in particular the ECM stiffness (Discher et al., 2005). Nearly two decades ago, a study was published which revealed how differently fibroblasts looked and moved on substrates with the same chemical properties but different stiffness (Pelham and Wang, 1997). Intriguingly, fibroblast locomotion and focal adhesion were regulated by the stiffness of their substrate. Simulated by these findings, diverse follow-up studies were published (Engler et al., 2004, 2006; Gilbert et al., 2010; Arulmoli et al., 2015; Sosale et al., 2015). In some instances mechanical properties of the microenvironment were observed to modify or even override chemical signals (Engler et al., 2006). Hence, it is meantime beyond doubt that the feedback of local ECM stiffness on the cell state has profound implications for development, differentiation, disease, and regeneration (Discher et al., 2005). However, the exact mechanism underlying stiffness sensing remains unclear (Tee et al., 2009; Moore et al., 2010; Franze et al., 2013; Humphrey et al., 2014). Mechanosensing of the environment is within microseconds followed by transmission of the gathered information to the cell nucleus to trigger a biochemical response in a physiological process known as mechanotransduction (Jaalouk and Lammerding, 2009; Wang et al., 2009). Cytoskeletal proteins, integrins, nesprins and the nuclear lamina are among the diverse proteins involved in the transmission process (Jaalouk and Lammerding, 2009; Wang et al., 2009). Mutation or misregulation of any proteins involved in mechanotransduction is closely associated with the onset and progression of diverse diseases ranging from muscular dystrophies to cardiomyopathies, cancer and neuropathies among others (Franze, 2013; Franze et al., 2013). Numerous studies highlight the importance of mechanotransduction for the nervous system thereby prompting a new branch of neurosciences termed neuromechanics (Franze, 2011). Diverse types of neurons and glial cells of the CNS have been shown to respond strongly to the stiffness of their substrate (Flanagan et al., 2002; Georges et al., 2006; Jiang et al., 2008; Rosenberg et al., 2008; Moshayedi et al., 2010). Substrate stiffness has profound influence on the morphology, development, networking, dynamics, behavior and function of neuronal and glial cells (Kostic et al., 2007; Previtera et al., 2010; Cellot et al., 2011; Fabbro et al., 2012; Tang et al., 2013; Zhang et al., 2014). Neuronal cells may even use stiffness gradients of their surrounding as a guidance for their migration, a process referred to as mechanotaxis (Franze, 2011). Unlike the CNS which certainly received some attention so far, the importance of mechanosensitivity for the PNS is just beginning to be recognized (Koch et al., 2012; Athamneh et al., 2015; Poitelon et al., 2016; Urbanski et al., 2016; Rosso et al., 2017).

## Schwann Cells

The principal glial cells of PNS are SCs. The generation of SCs is a multi-step process termed SC lineage (Mirsky and Jessen, 1996; Jessen and Mirsky, 2005). It starts from the migrating neural crest cells from the dorsal root ganglion, which develop first to SC precursors and then to immature SCs (Jessen and Mirsky, 2005). Immature SCs undergo a transition to two different cell types, myelinating and non-myelinating SCs. This decision of immature SCs on their fate as myelinating or non-myelinating is still puzzling (Jessen and Mirsky, 2005). All immature SC are held to share the same developmental potential and their decision to become myelinating or non-myelinating is determined by the axon they associate with Jessen and Mirsky (2005). It is well-known that only axons larger than ~1 μm in diameter are myelinated (Jessen and Mirsky, 2005) and that axonal Neuregulin-1 (NRG1) signaling is a prerequisite (Michailov et al., 2004; ffrench-Constant et al., 2004; Taveggia et al., 2005; Pereira et al., 2012; Heller et al., 2014). Nevertheless, the triggers for myelination and the resulting downstream signaling are still a matter of intense debate (Pereira et al., 2012). The two types of SCs play different roles in the PNS and both are pivotal for its maintenance (Jessen and Mirsky, 1999). The production and wrapping of myelin around axons permits fast conduction of signaling essential for the nervous system function. In addition to myelin production, SCs carry out numerous tasks that are indispensable for the development, physiological support, survival, protection and regeneration of the PNS (Jessen and Mirsky, 1999). Several of these functions in turn, are mediated by the basal lamina (Chernousov et al., 2008), whose production is a sequential process starting at the stage of immature SCs (Jessen and Mirsky, 2005).

## Implications of Schwann Cells Mechanosensitivity for PNS Development, Myelination and Regeneration

**Figure 1:**
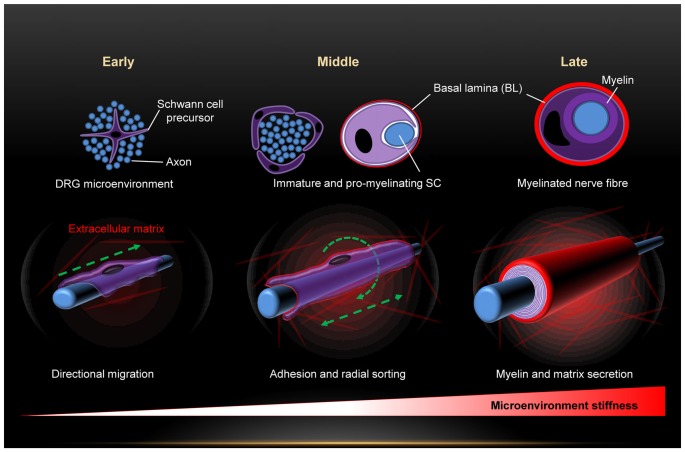
Dynamic change of the mechanical microenvironment of Schwann cells (SCs) during the developmental stages (early, middle and late) of the peripheral nervous system (PNS). Sequential build-up of basal lamina and several tissues progressively increases the microenvironment stiffness.

Exposure of SCs to mechanical force begins right from the early embryonic stages and is maintained throughout life. Among the major forces is the substantial increase in the stiffness of SCs microenvironment along their morphogenesis (Figure 1). This is due to progressive production of connective tissue and basal lamina which peaks in the stage of mature SCs (Jessen and Mirsky, 2005; Figure 1). Eventually, mature SCs are sandwiched between the ECM, the myelin and the axon, and surrounded by several connective tissues. Hence, maturation of SCs is paralleled by a dynamic change of the stiffness of their microenvironment. A recent work demonstrates with atomic force microscopy (AFM) measurements a consistent increase in the stiffness of peripheral nerves during development (Urbanski et al., 2016). Several lines of recent evidence demonstrate that SCs feel and respond to the stiffness of their substrate, and that mechanosensitivity plays key roles in SCs shape, adhesion, migration, differentiation, gene expression profiles and myelination (Gu et al., 2012; López-Fagundo et al., 2014; Fernando et al., 2016; Poitelon et al., 2016; Urbanski et al., 2016; Rosso et al., 2017). This strong impact of mechanosensitivity on SCs is of great importance for the PNS from biomedical and clinical aspects. Starting with PNS development, migration of SC precursors from embryonic DRGs is a fundamental step in PNS development. Adhesion is necessary for close interactions with outgrowing neurites in particular during the process of radial sorting for myelination of axons (Jessen and Mirsky, 2005; Brinkmann et al., 2008; Nave and Werner, 2014; Feltri et al., 2016). Differentiation and alteration of gene expression profiles are further critical steps during PNS development (Mirsky and Jessen, 1996; Jessen and Mirsky, 1999, 2005; Previtali et al., 2003; Pankov et al., 2005; Nodari et al., 2007; Leitman et al., 2011; Poitelon et al., 2016; Quintes et al., 2016). The morphology of SCs is of substantial physiological relevance for it strongly affects their protein expression levels and biochemical signaling (Migliore and Shepherd, 2005; Pankov et al., 2005; Nodari et al., 2007; Mitchel and Hoffman-Kim, 2011). For instance, SCs switch their morphology from elongated lamellae to the extension of radial lamellae in order to switch from migration to axonal sorting and myelination (Nodari et al., 2007). This morphological transformation is triggered by a switch in β1 integrin-activated Rac signaling (Nodari et al., 2007). Regeneration of the PNS upon injuries requires similar steps as in PNS development (Conforti et al., 2014). Transection of nerves following injury causes changes in the nerve stump distal to the injury that are collectively referred to as Wallerian degeneration (Jessen and Mirsky, 2008). Hallmarks are axon death, macrophages invasion, breakdown of myelin sheaths and transient phase of SCs proliferation, and the molecular expression pattern of SCs is reversed from that characteristic of mature myelinating and non-myelinating back to one characteristic of immature state (Jessen and Mirsky, 2008). Hence, SCs undergo dedifferentiation during certain PNS nerve injuries and will have to repeat major steps they take during PNS development. Investigation of PNS mechanosenstivity will not only substantially refine our insufficient understanding of PNS development but may also prove invaluable for clinical aid of nerve regeneration. The importance of including mechanosensitivity as a new key parameter in the design of nerve grafts is highlighted in recent works (Gu et al., 2011, 2012; Rosso et al., 2017). Under the influence of neurotrophic factors, the ECM and SCs, the proximal stump starts to sprout and elongate new axons in an attempt to regenerate the nerve. Elongation is mediated by the growth cones and the regeneration process occurs at a modest rate of 2–5 mm/day, which means that significant injuries may take very long (many months) to heal if at all (Gu et al., 2011). Hence, the regeneration rate essentially runs a very tight race against the Wallerian degeneration (Gu et al., 2011). Medical intervention must therefore be swift and the increase of the regeneration rate is decisive for success. This is where efficient scaffolds come in to tip the scales in favor of nerve healing. Mechanosensitivity is gaining particular attention in nerve scaffolds bioengineering (Gu et al., 2011). It is also coming in the focus of myelination research. A wealth of information has been obtained about myelination mechanisms so far, but some questions remain open (ffrench-Constant et al., 2004; Michailov et al., 2004; Nave and Salzer, 2006; Brinkmann et al., 2008; Pereira et al., 2012; Poitelon et al., 2016; Urbanski et al., 2016). The evidence is compelling that neuronal growth factor NRG1 type III signals information about the size of the axons to SCs, and thus plays a key role in the determination of the myelination fate of axons (ffrench-Constant et al., 2004; Michailov et al., 2004; Pereira et al., 2012; Heller et al., 2014). NRG1 effect is mediated by interaction with the glial ErbB2 receptors (Brinkmann et al., 2008; Mei and Nave, 2014). Myosin II activity and vimentin are further factors involved in PNS myelination (Wang et al., 2008; Leitman et al., 2011; Triolo et al., 2012). Nevertheless, the triggers for myelination and the resulting downstream signaling are still debated (Pereira et al., 2012; Nave and Werner, 2014; Feltri et al., 2016). Recent works demonstrate that PNS myelination is highly sensitive to the mechanical stiffness of the substrate (Poitelon et al., 2016; Urbanski et al., 2016). Yap and Taz, effectors of the Hippo pathway, which are known to integrate chemical and mechanical signals in cells (Dupont et al., 2011), control mechanosensitive PNS myelination (Poitelon et al., 2016).

Yap and Taz are TEAD transcriptional activators. A recent study demonstrates that mechanosensitive activation of Yap and Taz in SCs is followed by their regulation of both SCs proliferation and transcription of basal lamina receptor genes, two actions necessary for radial sorting of axons and subsequent myelination (Poitelon et al., 2016). The same study also shows that Yap and Taz are required for the expression of integrin α_6_ and β-dystroglycan in SCs, and that laminin-binding integrins and dystroglycan are important downstream targets of Yap, Taz and TEAD transcription.

## Implications of Schwann Cells Mechanics for PNS Function and Neuropathies

Given the absence of a hard mechano-protective physical barrier around peripheral nerves, we put forward the following hypothesis: Inherent structural elements of peripheral nerves impart biomechanical resilience and integrity to nerves. To test this hypothesis, we designed experimental strategy based on simultaneous combination of atomic force with confocal microscopy (Rosso et al., 2014). Mechanical resilience and structural integrity of large and small caliber peripheral myelinated nerve fibers were investigated both upon exposure to three incremental loading forces as well as after removal of the forces. Tested adult myelinated peripheral nerve fibers withstand striking mechanical compression reversibly without apparent damage or loss of biomechanical and structural integrity. Loading forces gradually stepped up to 75 nN compress the fibers. However, even the greatest force which causes substantial compression of fibers fails to disrupt their structural and functional integrity. This is striking taking into consideration the fact that the local loading force is exerted by a fairly sharp and rigid AFM tip which may be likened to a needle. The “needle”, in turn, generates a very high local pressure with respect to the relationship between pressure, force and area. Far lower forces (<2 nN) are generally needed to inflict substantial structural damage on cells when mechanically probed by a pyramidal AFM tip (Chiou et al., 2013). Hence, it is obvious that there is a natural mechanical protection of nerve fibers which makes up for the absence of a rigid physical barrier as in the CNS. It is also clear that mechanical resilience of peripheral nerve fibers must come from their intrinsic structural elements. Interestingly, neither the myelin sheaths nor the cytoskeleton significantly contribute to the biomechanical resilience of the nerve fibers but the fairly thin and flexible SCs basal lamina (Rosso et al., 2014). SCs basal lamina components typically include laminins, collagens and proteoglycans among other components (Court et al., 2006). Chemical digestion of collagens with collagenase leads to mechanical destabilization of myelinated nerve fibers (Rosso et al., 2014). Light mechanical compression of collagen-free fibers immediately results in a loss of their physiologically crucial tightness and in an irreversible structural deformation. The structural arrangement of the basal lamina plays a central role in mediation of the mechanoprotective effect (Rosso et al., 2014). A change of the basal lamina structural arrangement from a networked to a parallel orientation pattern, as observed in neuropathic *Pmp22*^−/−^ mice, is associated with a loss of the biomechanical integrity of nerve fibers; *Pmp22*^−/−^ mice are among the animal models to study the relationship between peripheral myelin protein 22 (PMP22) and the pathogenesis of hereditary PNS neuropathies (Adlkofer et al., 1995). The flexibility and particular structural arrangement of the thin basal lamina make it act much like a shock absorber or an armor, which reduces stress and tension on nerve fibers. Large mechanical forces exerted upon fibers are locally absorbed and dissipated thereby preventing fibers from mechanical vulnerability in a highly effective manner. PMP22 seems to contribute significantly to the mechanoprotective action of SCs basal lamina (Rosso et al., 2014). As a matter of fact, defects or mutations in PMP22 are a major cause of several hereditary peripheral neuropathies (Chance et al., 1993; Nicholson et al., 1994; Sereda et al., 1996; Suter and Scherer, 2003; Meyer Zu Hörste and Nave, 2006; Fledrich et al., 2014). PMP22 is crucial for the sealing, tightening and stabilization of myelin through its involvement in the assembly of myelin junctions, such as tight junctions and adherens (Guo et al., 2014). In addition, it is engaged in proliferation, differentiation and death of SCs (Jetten and Suter, 2000; Sancho et al., 2001; Amici et al., 2007), and the mechanical support of nerves (Bai et al., 2010). PMP22-associated PNS diseases such as Charcot-Marie-Tooth 1A (CMT1A; Fledrich et al., 2014) and hereditary neuropathy with liability to pressure palsies (HNPP) show mechanical vulnerability. Nerves from CMT1A rat model, with overexpression of PMP22 (Pmp22, gas-3), are prone to mechanical stresses (Sereda et al., 1996). Nerves from HNPP mouse model (*Pmp22*^+/−^) fail to recover from a mechanically induced 60%–70% conduction block even after days (Bai et al., 2010), and they lose their physiologically crucial tightness to small and large molecules (Guo et al., 2014). Trembler-J mice (Tr-J/^+^, mouse model for CMT1A) with a point mutation in *Pmp22*, exhibit impaired mechanical integrity. In *Pmp22*^−/−^ mice myelination is delayed and the basal lamina is loose (Amici et al., 2006), which indicates altered interactions of SCs with their basal lamina. Indeed, the levels of beta4 integrin, which is engaged in linking SCs with the basal lamina, are severely reduced in *Pmp22*^−/−^ mice (Amici et al., 2006). Together, the evidence is growing that PMP22 is not only important for the sealing of myelinated nerve fibers but also for their protection from mechanical stresses. PMP22 acts in concert with the basal lamina to provide PNS nerves with substantial biomechanical, structural and functional integrity. Myelin tightness and the ability of nerve fibers to withstand mechanical stresses and recover from compression are of paramount importance to ensure the physiologically critical high conduction velocity of myelinated nerves. Consequently, deficiencies, dysfunction or mutations in PMP22 and/or the basal lamina should have direct implications for PNS pathologies.

## Conclusion

SCs provide crucial biomechanical support to the PNS at the single nerve fiber level by a concerted action of the basal lamina and PMP22. PNS neuropathies linked to SCs, basal lamina and PMP22 should be explored in depth from biomechanical aspects. Comprehensive investigation of SCs mechanosenstivity will substantially refine our understanding of PNS development, physiology and diseases. At the same time, it holds promise to significantly advance the worldwide urgent field of PNS injuries treatment strategies.

## Author Contributions

GR and VS designed the manuscript and the figure and wrote the final version. PY was involved in the medical and pathological aspects discussed throughout the manuscript. All three authors approved the final version of the manuscript.

## Conflict of Interest Statement

The authors declare that the research was conducted in the absence of any commercial or financial relationships that could be construed as a potential conflict of interest.
